# The convergence of regenerative medicine and rehabilitation: federal perspectives

**DOI:** 10.1038/s41536-018-0056-1

**Published:** 2018-10-10

**Authors:** L. F. Rose, E. J. Wolf, T. Brindle, A. Cernich, W. K. Dean, C. L. Dearth, M. Grimm, A. Kusiak, R. Nitkin, K. Potter, B. J. Randolph, F. Wang, D. Yamaguchi

**Affiliations:** 1 Clinical and Rehabilitative Medicine Research Program, U.S. Army Medical Research and Materiel Command, Fort Detrick, MD USA; 2 Department of Veterans Affairs, Office of Research and Development, Washington DC, USA; 3 National Center for Medical Rehabilitation Research, Eunice Kennedy Shriver National Institute for Child Health and Human Development, National Institutes of Health, Bethesda, MD USA; 4 Tissue Injury and Regenerative Medicine Project Management Office, U.S. Army Materiel Development Authority, U.S. Army Medical Research and Materiel Command, Fort Detrick, MD USA; 5 Extremity Trauma and Amputation Center of Excellence, Walter Reed National Military Medical Center & Uniformed Services University of the Health Sciences, Bethesda, MD USA; 6 Disability & Rehabilitation Engineering and Engineering of Biomedical Systems Programs, National Science Foundation, Alexandria, VA USA; 7 National Institute of Arthritis and Musculoskeletal and Skin Diseases, National Institutes of Health, Bethesda, MD USA; 8 Veteran’s Administration, Greater Los Angeles Healthcare System, Los Angeles, CA USA

## Abstract

Regenerative rehabilitation is the synergistic integration of principles and approaches from the regenerative medicine and rehabilitation fields, with the goal of optimizing form and function as well as patient independence. Regenerative medicine approaches for repairing or replacing damaged tissue or whole organs vary from utilizing cells (e.g., stem cells), to biologics (e.g., growth factors), to approaches using biomaterials and scaffolds, to any combination of these. While regenerative medicine offers tremendous clinical promise, regenerative rehabilitation offers the opportunity to positively influence regenerative medicine by inclusion of principles from rehabilitation sciences. Regenerative medicine by itself may not be sufficient to ensure successful translation into improving the function of those in the most need. Conversely, with a better understanding of regenerative medicine principals, rehabilitation researchers can better tailor rehabilitation efforts to accommodate and maximize the potential of regenerative approaches. Regenerative rehabilitative strategies can include activity-mediated plasticity, exercise dosing, electrical stimulation, and nutritional enhancers. Critical barriers in translating regenerative medicine techniques into humans may be difficult to overcome if preclinical studies do not consider outcomes that typically fall in the rehabilitation research domain, such as function, range of motion, sensation, and pain. The authors believe that encouraging clinicians and researchers from multiple disciplines to work collaboratively and synergistically will maximize restoration of function and quality of life for disabled and/or injured patients, including U.S. Veterans and Military Service Members (MSMs). Federal Government agencies have been investing in research and clinical care efforts focused on regenerative medicine (NIH, NSF, VA, and DoD), rehabilitation sciences (VA, NIH, NSF, DoD) and, more recently, regenerative rehabilitation (NIH and VA). As science advances and technology matures, researchers need to consider the integrative approach of regenerative rehabilitation to maximize the outcome to fully restore the function of patients.

## Introduction

Regenerative rehabilitation is at the intersection of regenerative medicine and rehabilitation research: using the principles of rehabilitation sciences to maximize the outcome in the treatment of disabling conditions by regenerative medicine. While regenerative medicine approaches provide unique opportunities to regenerate, repair, and/or replace various tissues and organs, these approaches often fall short in the long-term treatment of chronic, disabling conditions whether in traumatically injured U.S. Veterans and Military Service Members (MSMs) or in the broader civilian population. Regenerative medicine or rehabilitation approaches provide a foundation for the restoration of tissue architecture, promotion of organ function, reduction of disability, or improvement of quality of life. However, it is the combination of both approaches working synergistically that can optimize or maximize the functional outcome of the individual. As an example, post-traumatic osteoarthritis (PTOA) is the single leading cause of failure to return to active duty after injury in the armed services.^1^ In the majority of these cases, the PTOA could be directly linked to articular fracture from explosions. The standard of care for PTOA is management of pain and eventual joint replacement. Rehabilitative strategies and therapeutics have not demonstrated success at preventing or resolving PTOA. A regenerative medicine strategy would entail regeneration of the articular cartilage, and while numerous studies are working on this, none are yet FDA approved. A regenerative rehabilitation technique might involve the pairing of an articular cartilage regeneration therapy with a rehabilitation therapy to yield synergistic improvements over either therapy alone.

**Fig. 1:**
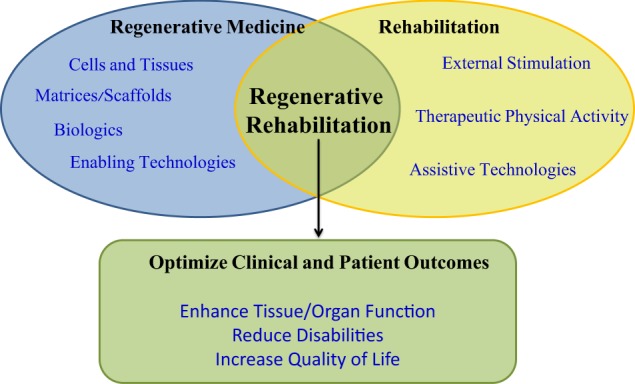
The goals of the regenerative rehabilitation approach are to synergize regenerative medicine approaches with rehabilitation techniques to enhance the clinical outcomes of the patient. Federal funding agencies understand the synergies between the basic biological approaches and clinical interventions that would improve tissue and/or organ function which could improve outcomes for patients

Several research programs (Table 1) have begun to combine both approaches to maximize the potential of each discipline, re-training the body and brain to facilitate recovery of function mediated by engineered tissues (Fig. 1). The federal agencies funding regenerative medicine and rehabilitation research would like to stimulate collaboration between researchers and clinicians from different disciplines to utilize the activity-mediated principles of rehabilitation medicine to enhance the potential of regenerative approaches to restore function. Towards this goal, each agency has provided a brief statement highlighting the areas of research interest.

- The Department of Defense (DoD) funding in medical research is driven by the unique needs of military clinicians treating combat injuries. Gaps in a clinician’s ability to address those injuries drives written requirements, around which research directives are built. The overall goal is to return Wounded Warriors to duty or improve their functionality and quality of life for transition to civilian life. The convergence of regenerative medicine and rehabilitation fields is recognition that neither discipline alone can fully address the injuries sustained by Service Members.
- The department of veterans affairs (VA) regenerative medicine program incorporates innovative and multidisciplinary approaches to solve complex tissue repair and replacement challenges relevant to veterans for a multitude of diseases and conditions with stem cell and bioengineering approaches. The overall goal is to develop regenerative approaches combined with unique rehabilitative, post-procedural care to maximize recovery of function while minimizing adverse side effects, with the ultimate goal of returning the veteran to a productive and independent life. This “pathway” to successful translation of cell therapies is being put into practice as VA evolves and expands its breadth of therapeutic options.
- The National Institutes of Health (NIH) support a range of research on molecular, cellular, and bioengineering approaches to replace tissue structure and to promote adaptation and recovery of function in support of individuals with disabilities. In recent years, the field of regenerative rehabilitation has been stimulated by advances in stem cell biology, bioengineering and nanomedicine, biophysical interactions, activity-mediated processes, and a greater understanding on the systemic effects of exercise. Translation of this research into clinical applications requires collaborations among a variety of basic and clinical fields with a clear focus on functional goals and practical therapeutic solutions.
- The National Science Foundation (NSF) Engineering of Biomedical Systems (EBMS) program supports the development of engineered hybrid systems for the treatment of disease or injury. Additionally, EBMS supports the development and validation of computational or experimental models that can elucidate the underlying physiological mechanisms that benefit from a rehabilitation approach to regenerative medicine and optimize these interventions. The Disability and Rehabilitation Engineering (DARE) program supports research in all areas of engineering that will enhance the quality of life for individuals with disabilities. Thus, regenerative rehabilitation research approached from an engineering point-of-view would also be of interest to this program. Based on its mission, NSF supports foundational research in these areas through proof of concept, transitioning to other agencies for further development.

**Table 1:** Examples of regenerative rehabilitation

*Regenerative rehabilitation: skeletal muscle*
Early translational work in humans to incorporate extracellular matrix bioscaffolds to repair volumetric muscle loss demonstrate the promise of human applications of regenerative rehabilitation.^21^ Through a series of case reports, investigators documented the after transplant of an extracellular matrix to severely damaged muscle tissue, thirteen subjects demonstrated significant gains on functional measures, increased gains in muscle tissue growth, increased presence of neurogenic growth in the transplant area, and improved nerve conductivity in the affected area.^22^ Though no controls were available, all candidates had received standard of care debridement and other procedures with no functional or neurogenic response. This extension of findings from animal models, suggests muscle loading following implantation is central to regenerating muscle tissue that is supportive of increased function.
*Regenerative rehabilitation: brain plasticity*
Boninger et al.^23^ suggest the combinatorial use of robotics, stem cell therapies, and brain computer interfaces for treatment of hemiparesis after stroke. Although modest efficacy has been observed in some cell therapy studies,^24–28^ it is clear that additional therapies are needed. Boninger et al propose the use of robots to deliver well-defined and reproducible forms of exercise therapy. Although optimal rehabilitation strategies remain unknown, many rehabilitation clinicians believe that stroke patients rarely receive sufficient therapy for optimal recovery. The combinatorial use of cell therapies and robotic-delivered therapy may enhance and optimize outcomes.
*Regenerative rehabilitation: bone*
Rehabilitation and regeneration following bone fracture and segmental bone defect have both been explored separately. Rehabilitation strategies including weight bearing timing, ultrasound therapy, and low magnitude mechanical signals have all been evaluated clinically.^29–31^ Cellular, genetic, and biologic regenerative strategies have been successfully demonstrated as well.^32^ Research involving the combination of rehabilitation and regenerative medicine strategies in vitro and in animal models has emerged with promising results.^33,34^ Continued research on the combination of rehabilitation and regenerative medicine strategies in bone healing may show optimized outcomes.

### Drivers

Federal interest in regenerative rehabilitation stems from clinical needs. As regenerative medicine therapies reach the clinic, outcomes suggest cellular and tissue focus may be insufficient to address clinical needs. Significant advances in medicine are improving survival from catastrophic injuries, but often fail to fully restore function and thus may benefit from regenerative rehabilitation. For example, the rate of extremity injuries (55%) in Operation Iraqi Freedom and Operation Enduring Freedom was similar to that observed during World War II.^2^ However, high-energy improvised explosive devices used in the recent conflicts resulted in more severe injuries with limited tissues suitable for salvage or reconstruction.^3,4^ Over the last decade, the ability to save the lives of these severely injured MSMs has improved but resulted in a new population with major reconstructive and rehabilitative needs, some of which exceed the capabilities of conventional medicine. The added complexity of penetrating trauma, blunt injuries that damage multiple surrounding tissues, and burn components complicate the treatment, and new therapies such as dermal substitutes, tissue matrices and new surgical strategies are often inadequate to deal with these more complicated injuries.^5–7^

In addition, MSMs experience training and overuse injuries at higher rates than their civilian counterparts,^8^ such as low back pain, knee and hip osteoarthritis, ankle instability, and rotator cuff injuries. Surgical and rehabilitation treatment strategies are often insufficient, resulting in lost duty days and a significant reduction in force readiness.^9^

The concept of regenerative rehabilitation is thus driven by the demographics of injured MSMs: younger, healthier, with less co-morbidity, and long life expectancy after injury. This population is far less amenable to a treatment that restores form without fully restoring function. Although reconstructive surgeries, regenerative medicine approaches, or prosthetics and orthotics may each contribute to a level of basic functionality, only these therapies combined with a robust rehabilitative strategy can provide the level of function desired by these patients (Fig. 1). In short, regenerative rehabilitation therapies must not simply be additive in nature, but must be synergistic.

Although acute injuries outside of the Military Health System are generally less severe and extensive, patients can still benefit from regenerative rehabilitation, including the veteran population with continuing care after extensive trauma or chronic conditions due to aging. Extended regenerative rehabilitation may also apply to conditions such as chronic inflammation, surgical treatment of heterotopic bone formation, or revisions of previous vascular reconstructions. Complications from chronic inflammation such as bone loss, fibrotic scar formation, loss of dexterity, and fine motor function, as well as loss or disability of skin appendages, such as sweat glands, can also make rehabilitation of the original reconstructed or regenerated tissue more difficult. The civilian healthcare sector faces a combination of the challenges delineated in military and veteran health care. The U.S. civilian population also incurs a host of traumatic injuries and diseases that lead to physical disability: burns, falls, automobile accidents, sports injuries, cancer, diabetes, cardiovascular disease, and degenerative neuromuscular disorders. Other diseases such as cancer, diabetes, and vascular conditions may lead to amputation of extremities that require ongoing care in the veteran population, as opposed to active duty military. The authors of this article believe that regenerative rehabilitation will better meet these clinical needs than regenerative medicine or rehabilitation alone.

### Considerations for the federal research enterprise

DoD medical research focuses on active MSM as well as those who have not yet been transitioned to the VA but will not likely return to active duty. Ultimately, the DoD’s interests in this space hinge upon the promise of therapies to return critically injured Warfighters to full function and deployability. The extensive trauma to the extremities seen in recent conflicts has resulted in comparatively extensive investments in regenerative medicine. However, the promise of regenerative medicine to fully restore MSMs is yet to be realized. These MSMs ultimately transition to the VA, joining a population of older veterans with a wider age range and scope of medical conditions. NIH’s broader mission covers the entire population from children to adults, men and women, and ethnically and socioeconomically diverse groups, and includes a wide range of regenerative strategies, such as vision restoration; dental and maxillofacial reconstruction; muscle and skin regeneration, and repair and regeneration of other organs and tissues. The NSF funding in this area focuses on foundational research that advances engineering and biomedical science, as appropriate to the program. Engineering research, by its nature, involves application and keeps in mind future translation. While NSF does not fund large scale clinical studies, feasibility studies involving human volunteers can be supported if appropriate to the research objectives. These mission differences allow consideration of variations in the safety, efficacy, and effectiveness requirements across health systems, throughout the lifespan and with differing degrees of concomitant or comorbid conditions. The ability of rehabilitation therapies to improve upon the outcomes currently achievable by regenerative medicine therapies provides hope for this diverse population to continue in service or to live fuller, more active lives with reduced healthcare requirements. Given the overlapping needs among the DoD, the VA, the NIH, and the NSF, the commercial market applicability may be expanded if the technologies and concomitant therapies are proven generalizable, safe, and effective.

### State-of-the-science of regenerative rehabilitation

Regenerative rehabilitation is built first on the foundation established in regenerative medicine. Current approaches in regenerative medicine involve the use of cells and/or biologics with or without scaffolding materials with the goal of replacing injured or lost tissue resulting from trauma or disease. However, the medical care process does not stop after transplantation of the cell or tissue construct in the clinic. Therefore, preclinical models need to take into account the role of rehabilitation post-transplantation. For example, the consequences of central nervous systems injuries illustrate the need for collaboration in clinical care and research in regenerative medicine and rehabilitation. Trauma to the central nervous system not only involves nerve cells but the peripheral targets these cells innervate, including internal organs and the musculoskeletal system. Rehabilitation prior to regenerative therapies can set the stage for improved recovery by pre-conditioning the individual, while post-transplant physical activity may help cells to integrate and form appropriate connections with the host tissue. Activity-dependent performance of tasks not only shapes behaviors but also strengthens synapses and promotes neuronal plasticity.^10,11^ In general, the timing, dosing, and duration of rehabilitation strategies for neurologic or musculoskeletal injuries varies, and their optimal integration with regenerative therapies is an area in need of further study (Table 2).

**Table 2:** Research interests and contact information for contributing institutions

National Institutes of Health (NIH) [in rank order of current rehabilitation research spending]	
Program official contacts	Rehabilitation research interests
1. National Institute of Neurological Disorders and Stroke (NINDS)	
Dr. Daofen Chen: daofen.chen@nih.gov Dr. Lyn Jakeman: lyn.jakeman@nih.gov Dr. Pat Frost Bellgowan: patrick.frostbellgowan@nih.gov Dr. Scott Janis: JanisS@ninds.nih.gov Website: http://www.ninds.nih.gov/	Understanding the fundamental mechanisms and evidence for effectiveness of rehabilitation on progression, neural plasticity and recovery of function in animal models or human subjects with neurological disorders or disease, or following injury to the brain, spinal cord or peripheral nervous system
	Research on the physiological mechanisms of environmental, socioeconomic, and demographic variables and disparities on effectiveness of rehabilitation interventions for individuals with neurological conditions
	Research on the effective delivery and outcome assessment of rehabilitation interventions for individuals with neurological conditions across the lifespan and around the world
	Precision based medicine research and identification of markers that inform mechanistic underpinnings and/or biological targets of action for neurorehabilitation therapies
	Development and use of nervous system stimulation and recording devices and sensors that can detect responses or influence the activity of the nervous system for improved diagnosis and/or functional recovery
	Approaches, tools and resources to improve the rigor and predictive power of preclinical, observational, and clinical studies in the area of neurorehabilitation
	Exploratory and definitive clinical trials of rehabilitation interventions at the stage appropriate for the level of evidence and burden of disease or disability
2. National Institute of Deafness and Communication Disorders (NIDCD)	
Dr. Lana Shekim: shekiml@nidcd.nih.gov Website: www.nidcd.nih.gov	Research to improve hearing healthcare (HHC) https://www.nidcd.nih.gov/research/improve-hearing-health-care
	Studies on the rehabilitation of neurologic communication disorders (aphasia, dysarthria, and apraxia of speech)
	Studies on neuromodulation in conjunction with behavioral therapy, for example, in management of tinnitus or in verbal expression
	Research on augmentative and alternative communication (AAC) in conjunction with brain-computer interface (BCI) for communication
3. *Eunice Kennedy Shriver* National Institute for Child Health and Human Development (NICHD) National Center for Medical Rehabilitation Research (NCMRR, within NICHD)	
Dr. Alison Cernich: alison.cernich@nih.gov **Dr. Ralph Nitkin:** nitkinr@mail.nih.gov Website: www.nichd.nih.gov www.nichd.nih.gov/about/org/ncmrr	Pathophysiology and management of chronically injured nervous and musculoskeletal systems (including stroke, traumatic brain injury, spinal cord injury, and orthopedic conditions)
	Repair and recovery of motor and cognitive function
	Functional plasticity, adaptation, and windows of opportunity for rehabilitative interventions
	Rehabilitative strategies involving pharmaceutical, stimulation, and neuroengineering approaches, exercise, motor training, and behavioral modifications
	Pediatric rehabilitation
	Secondary conditions associated with chronic disabilities
	Improved diagnosis, assessment, and outcome measures
	Development of orthotics, prosthetics, and other assistive technologies and devices
4. National Institute on Aging (NIA)	
Dr. Lyndon Joseph: josephlj@mail.nih.gov Website: www.nia.nih.gov	Exercise
	Physical therapy
	Pain management
	Mobility
	Gait
	Technology
	Robotics
5. National Cancer Institute (NCI)	
Dr. Ann O’mara: omaraa@mail.nih.gov Dr. Julia Rowland: rowlandj@mail.nih.gov Website: www.cancer.gov	The management of acute and chronic as well as late morbidities associated with cancer. The impact of cancer and its treatment on a wide variety of patient outcomes, such as fatigue, neurocognitive impairments, neuropathies, sexual function, general physical functioning, has been documented. However, because of the paucity of evidence
	The role of pre-habilitation as well as post-treatment rehabilitation in improving functional outcomes among cancer survivors. The unique contribution of rehabilitative services to cancer patient and survivors’ outcomes remains poorly understood. In addition, research is needed to test and evaluate the efficacy of different models of care delivery (timing, staffing, components, metrics for success) to determine the best way to integrate and deliver rehabilitative services across the cancer control continuum
6. National Institute of Arthritis and Musculoskeletal and Skin Diseases (NIAMS)	
Dr. Charles H. Washabaugh: washabac@mail.nih.gov Website: http://www.niams.nih.gov/	Examining the impact of physical activity levels on bone health and fracture risk and developing and testing strategies to promote bone health through exercise and physical rehabilitation programs
	Developing or modifying strategies, including preventive and rehabilitative approaches, to reduce the development of disability and functional limitation associated with OA onset and progression
	Exploring rehabilitation and physical-therapy strategies to reduce risk for impairment from OA progression
	Standardizing criteria for determining therapeutic effects of non-surgical interventions (such as drugs or rehabilitation strategies) to prevent or treat implant osteolysis
	Developing and validating pre-operative and post-operative rehabilitation strategies, especially for hip and knee replacement
	Applying physical medicine and rehabilitative strategies to soft-tissue injuries to restore maximal function
	Determining types and levels of exercise effective for minimizing progression of specific diseases and promoting restoration of musculoskeletal function
7. National Heart, Lung, and Blood Institute (NHLBI)	
Dr. Jerome Fleg: flegj@nhlbi.nih.gov Website: http://www.nhlbi.nih.gov/	Strategies to increase participation in cardiac and pulmonary rehabilitation programs
	Reduction of disparities in cardiac and pulmonary rehabilitation participation by women, minorities, the elderly, and low income individuals
	Development of new models for cardiac and pulmonary rehabilitation, including those incorporating telemedicine, fitness trackers, the Internet, and other novel technologies
8. National Eye Institute (NEI)	
Dr. Tom Greenwell: greenwellt@mail.nih.gov Dr. Cheri Wiggs: Cheri.Wiggs@nih.gov Website: https://nei.nih.gov/	Assistive devices for individuals with visual impairment
	Adaptive technologies and training specialized for low vision
	New technologies (including prostheses) for restoring vision to the visually impaired
	Rehabilitation strategies that address the special health problems and requirements of people with visual impairment
9. National Institute of Biomedical Imaging and Bioengineering (NIBIB)	
Dr. Grace C.Y. Peng: grace.peng@nih.gov Dr. Michael Wolfson: michael.wolfson@nih.gov Website: www.nibib.nih.gov	Novel methods and technologies to interact with a patient, including neural interfaces, physical interfaces, and sensory interfaces
	Novel sensors to monitor biomarkers of patient health and rehabilitation progress
	Novel prostheses and orthoses to facilitate rehabilitation and restoration of function
	Next generation computational models and intelligent methods for rehabilitation applications
10. National Institute of Nursing Research (NINR)	
Dr. Lois Tully: lois.tully@nih.gov Website: http://www.ninr.nih.gov/	Symptom and self-management strategies aimed at maintaining, improving, or restoring functional abilities and quality of life in individuals with functional impairments or disabilities resulting from injury, aging, or chronic illness
	Role of modifiable lifestyle and health behaviors on risk for initial disability (prevention) or on re-occurrence of the disability
	Informal caregiving of individuals with a disability
	Biological and psychosocial mechanisms underlying inter-individual variation in response to rehabilitation interventions
11. The National Institute of Diabetes and Digestive and Kidney Diseases (NIDDK)	
Dr. Teresa Jones: jonest@extra.niddk.nih.gov Website: http://www.niddk.nih.gov/	Improvements in the diagnosis and treatment of bowel, bladder, and erectile dysfunction
	Nutritional strategies to improve the quality of life for people with chronic kidney, gastrointestinal, endocrine, and metabolic diseases
	Improving the functional status of individuals with end-stage renal disease
	Gait, muscle, and peripheral nerve dysfunction secondary to diabetes
	Improving function in individuals with foot deformities or amputations of their lower extremities from the complications of diabetes
	The use of closed loop systems to compensate for the loss of beta cell function in type 1 diabetes
12. Office of Behavioral & Social Sciences Research (OBSSR)	
Dr. Bill Elwood: william.elwood@nih.gov Website: https://obssr.od.nih.gov/	Improves the synergy of basic through applied behavioral and social science research findings through projects that more precisely target individual and social mechanisms and processes that improve health and wellbeing
	Enhances measures, methods, and data infrastructure that encourage a more cumulative and integrated approach to social and behavioral aspects of rehabilitation research
	Facilitates the adoption of behavioral and social research findings in rehabilitation health research and practice
13. Office of Dietary Supplements	
Dr. Abby Gwen Ershow: ershowa@od.nih.gov Website: https://ods.od.nih.gov	Role of dietary supplements in maintaining and improving health and preventing chronic disease in individuals with mobility or other rehabilitation medicine issues
	Methods for assessing dietary supplement use by individuals with vision or hearing impairments or using assistive technologies
	Safety of nutritional, herbal, or botanical dietary supplements used by individuals with disabilities or participating in rehabilitation medicine activities
	Role of dietary supplements in meeting nutrient needs for optimal growth and health in children with mobility impairments or feeding difficulties

Department of Defense (DoD)	
Clinical & Rehabilitative Medicine Research Program (CRMRP)	
Regenerative medicine portfolio, neuromusculoskeletal (NMS) injury rehabilitation portfolio	
Program official contacts	Regenerative and rehabilitation research interests
Regenerative MedicineDr. Lloyd **Rose** lloyd.f.rose2.civ@mail.mil NMS Injury Rehabilitation Dr. Erik Wolf erik.j.wolf6.civ@mail.mil Website: https://crmrp.amedd.army.mil/	Efforts to replace or regenerate human cells, tissues or organs to restore or establish normal tissue function for regenerating traumatically injured tissues of the extremity and craniomaxillofacial injuries, burns and scarless wound healing, composite tissue transplantation
	Efforts directed towards optimal treatment, rehabilitation, and reintegration following service-related neuromusculoskeletal injury including: service-related acute and repetitive overuse injury management, limb loss rehabilitation and prosthetic management, and limb trauma rehabilitation and orthotic management

Department of veterans affairs office of research and development	
Basic Laboratory Research and Development (BLR&D) Service Trauma, Surgical and Musculoskeletal and Immune Disorders Portfolio Rehabilitation Research and Development (RR&D) Service Regenerative Medicine Portfolio RR&D Musculoskeletal and Co-morbidities Portfolio	
Program official contacts	Rehabilitation research interests
**BLR&D Dr. Kimberlee Potter** Kimberlee.potter@va.gov RR&D Musculoskeletal and Co- morbidities Dr. Timothy Brindle Timothy.brindle@va.gov RR&D Regenerative Medicine Dr. Audrey Kusiak Audrey.kusiak@va.gov Websites: www.research.va.gov	Research to develop strategies to replace or regenerate tissues or organs following traumatic injury or disease. The goal is to restore tissue function and increase the quality of life of veterans
	Rehabilitation and target-directed activity-based efforts post cell transplantation to strengthen and facilitate proper connectivity/integration of regenerative approaches
	Prehabilitation of subjects to maximize post-regenerative rehabilitation and timely recovery
	Long-term safety and efficacy of cell-based regenerative approaches
	Development of clinically relevant large animal models with scale-up and translational relevance

National Science Foundation (NSF)	
Engineering of Biomedical Systems Program (EBMS)	
Disability & Rehabilitation Engineering Program (DARE)	
Program official contacts	Regenerative and rehabilitation research interests
Dr. Michele Grimm mgrimm@nsf.gov Website: https://www.nsf.gov/div/index.js p?div=CBET	Fundamental and transformative research in: the development of validated models (living or computational) of normal and pathological tissues and organ systems that can support development and testing of medical interventions; the design of systems that integrate living and non-living components for improved diagnosis, monitoring, and treatment of disease or injury; and advanced biomanufacturing of three-dimensional tissues and organs
	Fundamental engineering research that will improve the quality of life of persons with disabilities through: development of new technologies, devices, or software; advancement of knowledge regarding normal or pathological human motion; or understanding of injury mechanisms

On the other hand, the inappropriate pairing of regenerative and rehabilitative approaches can have deleterious side effects (e.g., pain and spasticity following inappropriate peripheral inputs) and requires monitoring.^12^ Thus the foundation for regenerative rehabilitation can be laid by harnessing physical substrates to refine, direct, and mold components to the desired regenerative endpoint, while monitoring for adverse effect to restore and improve the functionality of the individual.^13^ As new regenerative medicine strategies enter human clinical trials, increased collaborations with rehabilitation clinicians will be important to optimize outcomes. It will also be important for rehabilitation clinicians to plan for potential changes in rehabilitation practice to accommodate regenerative medicine treatments.

### Maturity of the regenerative medicine field

The ultimate goal of regenerative medicine is to completely restore missing or damaged tissues to a level functionally and aesthetically indistinguishable from the pre-injury/diseased state. This entails not only regeneration of the specific tissues (such as nerve, muscle, bone, skin, and vasculature), but also integration of the tissues with each other and the healthy surrounding tissue. Apart from vascularized composite allotransplantation, reconstruction with like tissues through regeneration of autologous tissue is still a distant goal, and different tissues are at widely varying stages of maturity. Bone regeneration is most advanced, with several FDA-approved therapies currently in use clinically.^14^ Numerous technologies for regenerating skin are available, although final outcomes are still suboptimal.^15,16^ Several technologies to regenerate large diameter arteries are in development or in clinical trials.^17^ However regeneration of peripheral nerves is limited to 5–7 cm,^18^ and re-innervation of end organs is not guaranteed. Moreover, the ability to integrate those individual tissues into a functioning whole is only in the early stages of development. Variables such as cell source and host species play critical roles in the successful integration of the graft with the host. In the case of spinal cord injury, human cells transplanted into rodent spinal cord take over one year to mature^19^ and differentiate into glial cells and neurons, with functional recovery beginning more than one year after grafting. Despite hurdles such as graft integration and cell maturation, there is a trend among researchers and clinicians that clinical outcomes following the application of regenerative medicine technologies can be optimized by the addition of rehabilitation approaches.^20^

### Federal interest

The value of regenerative rehabilitation lies in the promise to patients of enhanced functional restoration and recovery from injuries and in the promise to the health care systems of reduced morbidity and healthcare utilization for those patients. Failed reconstructive or regenerative therapies require additional surgeries or supportive care that strain healthcare resources. Any reduction in the number or complexity of procedures, or any improvement in functional outcomes, reaps rewards to not only patients and their families, but also in healthcare outcomes (e.g., reduced hospitalizations, ability to return to work). Thus, the overall positive societal impact of successful regenerative rehabilitation treatments will be significant.

We recognize the need to promote rigorous research in regenerative rehabilitation. Collaborations between researchers in multiple disciplines of regenerative medicine, rehabilitation clinicians and engineers, and patients and their families, are essential to develop optimal systems and processes to support success in regenerative medicine therapies as they are developed and translated to clinical application. By involving all phases of treatment in the development of regenerative medicine therapies, it will be possible to improve outcomes and maximize the quality of life of Americans.

## Acknowledgments

The views expressed in this manuscript are those of the authors and do not reflect the official policy of the Department of Army or Navy, Department of Defense, Department of Veterans Affairs, National Science Foundation, National Institutes of Health, or U.S. Government.
